# Delayed axial flaps for coverage of a fourth degree scalp burn

**DOI:** 10.1093/jscr/rjac468

**Published:** 2022-10-21

**Authors:** S Moltaji, A El Khatib, A D Rogers

**Affiliations:** Division of Plastic, Reconstructive and Aesthetic Surgery, Department of Surgery, University of Toronto, Toronto, Canada; Centre Hospitalier de l’Universite de Montreal, Montreal, Canada; Division of Plastic, Reconstructive and Aesthetic Surgery, Department of Surgery, University of Toronto, Toronto, Canada; Ross Tilley Burn Centre, Sunnybrook Health Sciences Centre, Toronto, Canada

## Abstract

Although free tissue transfers may be required to cover wounds of the head and neck with bone involvement and exposure, options lower on the reconstructive ladder should still be considered during the planning process. We present a case of an elderly gentleman with a history of cardiovascular comorbidity and neck radiotherapy, who sustained a deep flame burn injury to his scalp. Two delayed axial flaps, based on the superficial temporal and supraorbital arteries respectively, were used to obtain durable coverage of this complex wound.

## INTRODUCTION

Most full thickness burn injuries, including those of the head and neck region, can be effectively covered with split thickness skin grafts. When there are defects with exposed or involved underlying bone, a flap reconstruction may be required. Although the modern inclination is to reconstruct these with free tissue transfers, plastic surgeons have traditionally considered all the possible options as per the reconstructive ladder [1, 2].

The blood supply to the scalp, based on five paired vessels, is particularly robust, and extensive collateralization exists. This advantage is offset by the relative paucity of excess tissue, therefore requiring skin grafts to cover local flap donor sites in most extensive cases [3, 4]. Here, we present our experience obtaining local flap coverage of a deep scalp defect resulting from a burn injury in an elderly male patient.

## CASE REPORT

**Figure 1:**
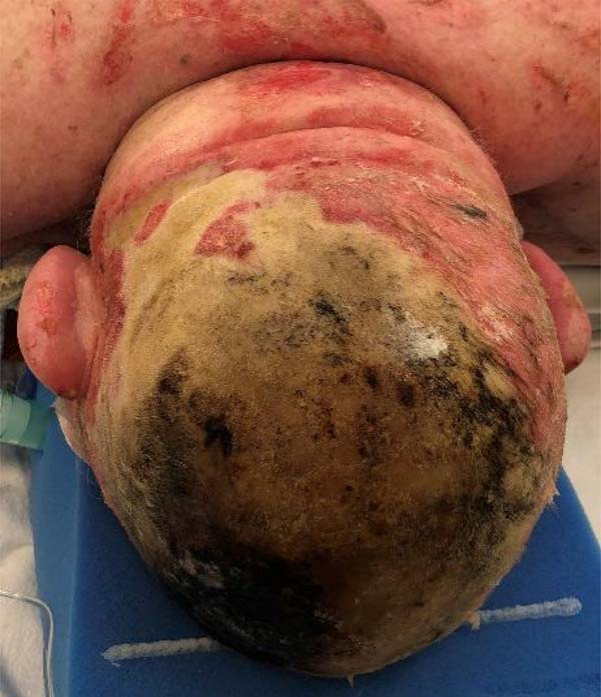
Burn Injury prior to excision, with patient in prone position.

**Figure 2:**
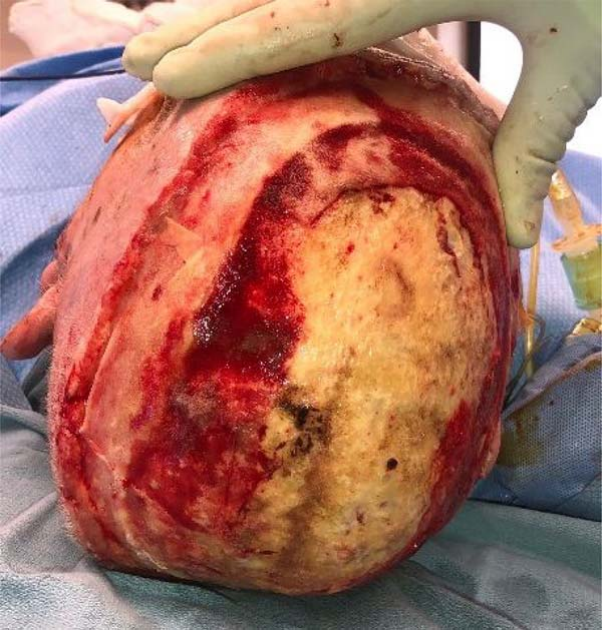
Following excision of scalp burn, down to and including outer table of the skull.

An 80-year-old male was referred to the regional burn centre with a full thickness scalp burn after falling into a woodstove (Fig. 1). He was assessed to have significant co-morbidities, including emphysema and coronary artery disease, and had received radiotherapy for squamous carcinoma of the palate. He underwent excision of his burns under general anesthesia the following day, necessitating burring down of the outer table over a large portion of the wound: moderate bleeding from the diploe was observed (Fig. 2). A negative pressure wound therapy with instillation and dwell™ technology (3M, Saint Paul, MN, USA) using Prontosan™ (BBraun, Melsungen, Germany) was applied.

At the subsequent surgery, a skin substitute (Integra Lifesciences, Princeton, NJ, USA) was applied. Dressings were continued for a period of 2 weeks, at which time it was apparent that the Integra™ was adherent around the periphery but not the 25 cm (long) by 15 cm (wide) central area. At this stage, our microsurgical team was consulted to consider free flap reconstruction but he was deemed a poor candidate, given his medical background, previous radiotherapy, the need for long vein grafts or a venous loop, and his advanced age.

**Figure 3:**
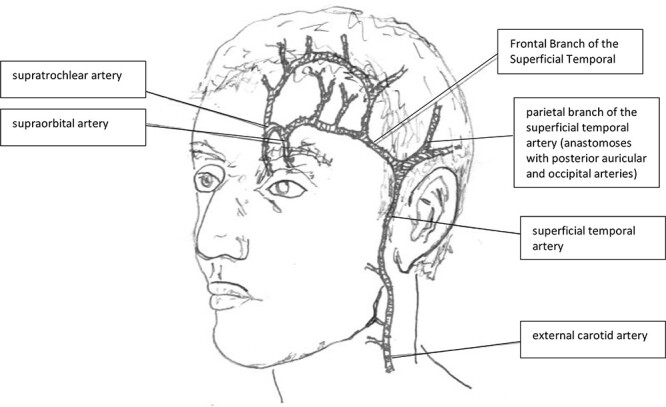
Surgical anatomy of the supraorbital and superficial temporal arteries, their origins and terminal branches relevant in this case.

**Figure 4:**
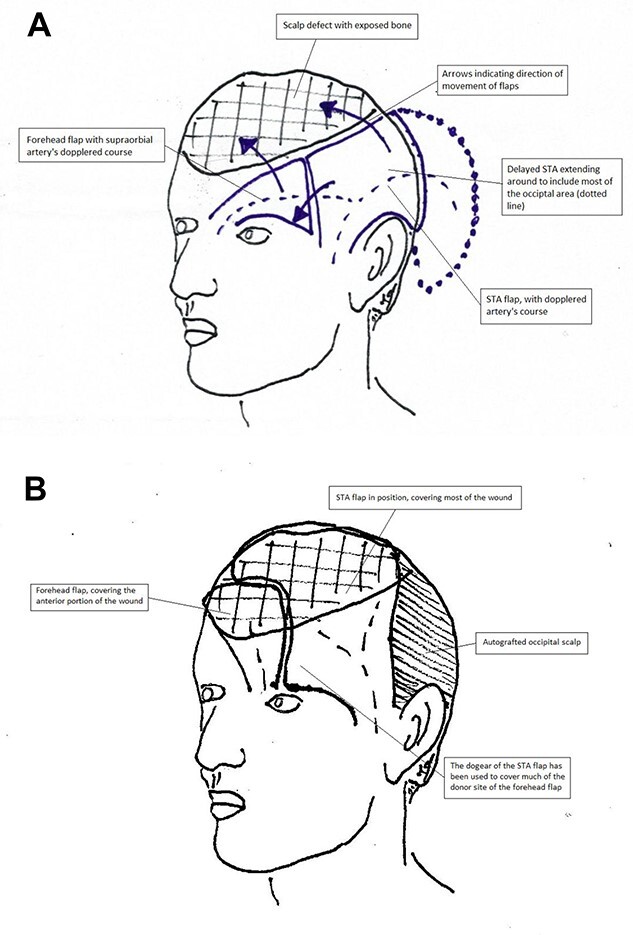
(**A**, **B**) Operative plan for coverage of the defect. The STA flap would transpose to cover the posterior part of the wound, whereas the forehead flap would cover the anterior. Note how the anterior portion of the STA flap which first creates a dog-ear anteriorly, then aids in the closure of the distal portion of the forehead flap donor site.

An alternate plan to utilize the remaining left temporal and occipital scalp as a delayed superficial temporal artery (STA) flap, in conjunction with left forehead flap, was devised. Figure 3 illustrates the vascular surgical anatomy and Fig. 4A and B outline the proposed plan. He understood that this would require several further surgeries under general anesthesia.

**Figure 5:**
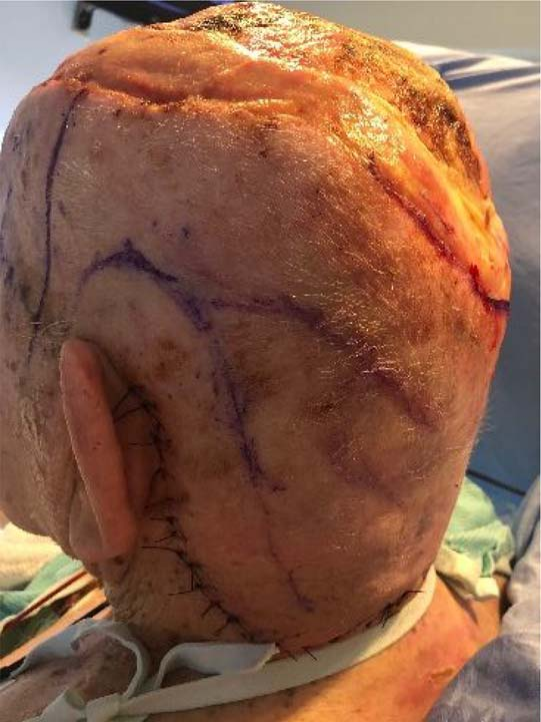
After the first delay procedure. Note the sutures around the distal extent of the delayed flap. The Doppler signal is marked with pen within the delayed STA flap.

During the first surgery, the course of the parietal branch of the left STA was dopplered and two branches were traced back into the occipital scalp. A bi-pedicled delay was undertaken, whereby the flap was designed to include as much of the unburnt occipital scalp as possible. The periosteum over this area was carefully protected during undermining and elevation. The distal incision was made at the subsequent surgery and the flap elevated up to the division of the STA into parietal and frontal branches (Fig. 5). The distal half of the flap appeared moderately congested with elevation, and was therefore replaced again.

**Figure 6:**
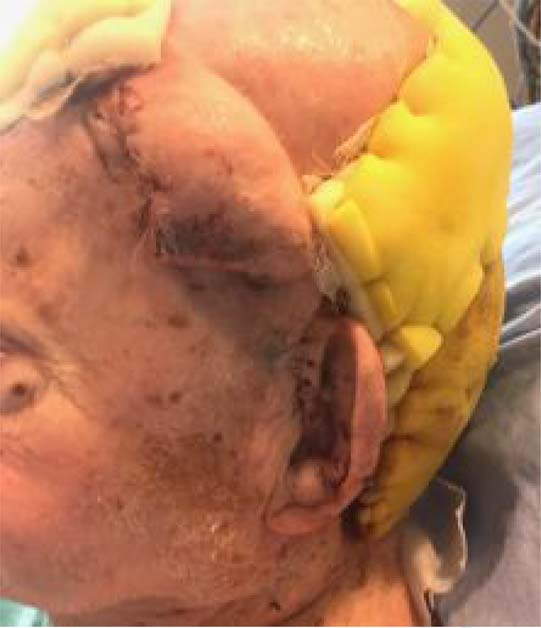
After initial inset of the STA flap, demonstrating large dog-ear, ultimately used to partially cover the donor site of the forehead flap.

**Figure 7:**
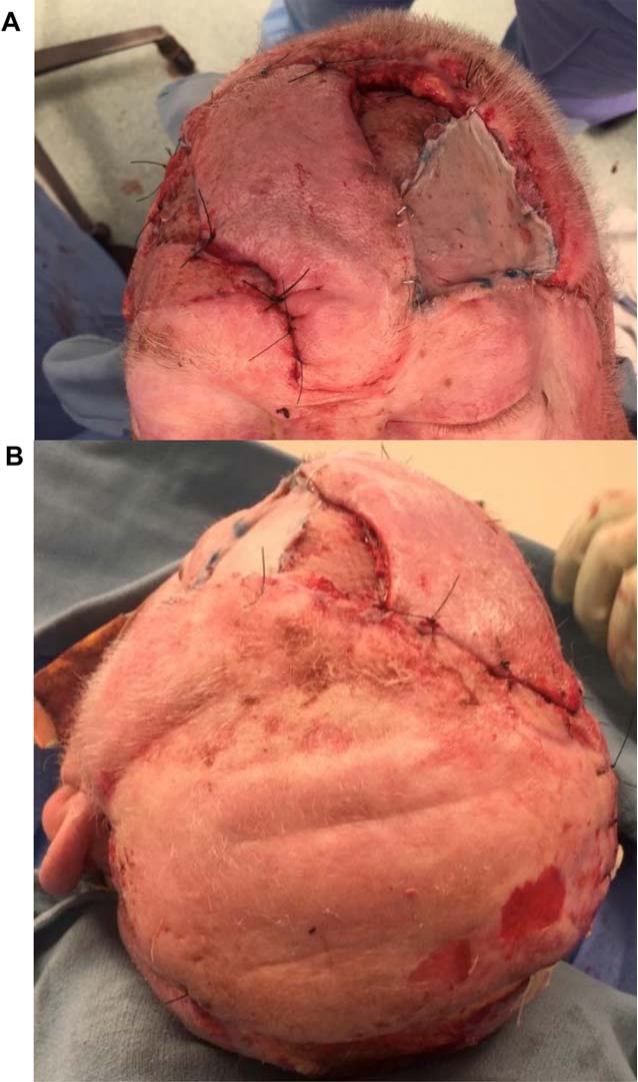
(**A**, **B**) Axial Forehead flap, with its donor site covered by the dog-ear of the STA flap and a sheet graft to the remaining area.

Five days later the flap was elevated and inset, and the occipital area was autografted with 2:1 split skin grafts obtained from his back. This flap covered ~75% of the defect, leaving an 8 by 5-cm area anteriorly (Fig. 6). Next a delayed axial forehead flap based on the supraorbital and supratrochlear vessels were raised. The lateral edges were incised and the flap undermined, leaving the distal pedicle intact (the dog-ear). One week later the patient was returned to the operating room to inset the forehead flap. This flap was then transposed into the defect, sutured to the anterior edge of the STA flap, and the remaining forehead donor site (not covered by the laid out dog-ear) was autografted. The ‘dog-ear’ created by the STA flap was utilized to cover the distal section of the donor site of the forehead flap (Fig. 7A and B).

**Figure 8:**
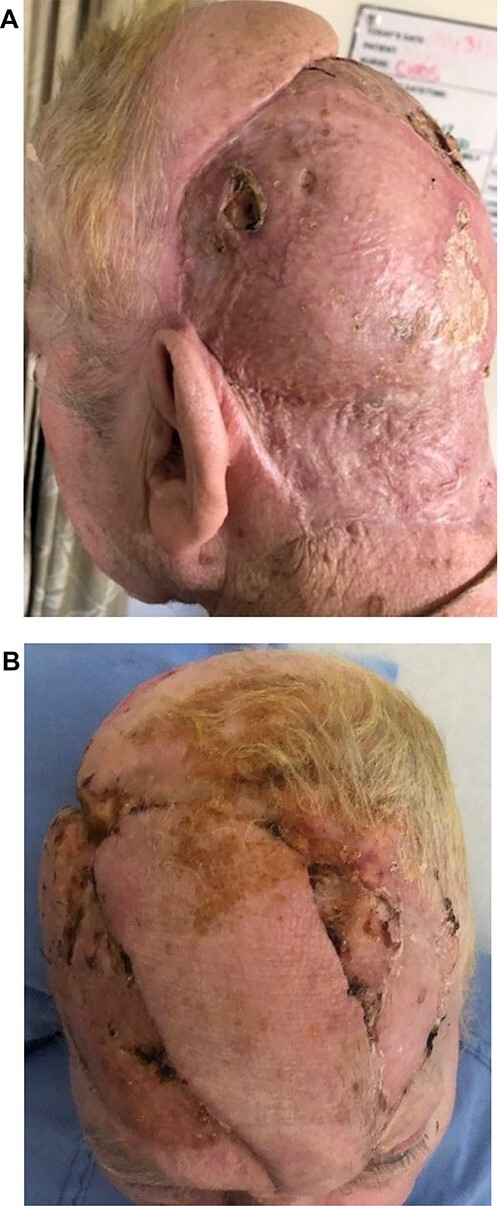
(**A**, **B**) Appearance on discharge from hospital.

In this way, the entire defect was covered with the two delayed axial flaps and skin grafts. Despite his co-morbidities, there were no perioperative complications. The grafts healed well and there was no flap loss (Fig. 8A and B). A period of wound care followed to the skin grafted areas, and all sutures were removed at 2 weeks. The patient has since returned home after a hospital stay of 2 months, regained his premorbid level of independence, and has had 2 years of follow-up without any sequelae.

## DISCUSSION

Although the reconstructive ladder advocates that the simplest flap should be considered first for the coverage of defects not amenable to primary closure or skin grafting, other factors taken into account are the number of procedures associated with each course, the resulting donor site and patient preference. Free tissue transfers are increasingly successful, and offer additional versatility when compared to local options [1, 2].

Here, we describe a case of a deep burn injury to a large portion of the scalp in an elderly gentleman. Free tissue transfer was not offered, owing to his cardiovascular risk profile, a history of radiotherapy of the neck, the need for a large flap with vein grafts, and therefore a significant perceived risk of flap loss [5–8]. Here, two delayed local flaps, along with autografts to donor sites and peripheries, were utilized for reconstruction in multiple stages. Fortunately, despite being exposed to the risk of multiple surgeries, our elderly patient ultimately obtained stable coverage with local flaps without perioperative morbidity.

The literature regarding deep scalp burns emphasizes the need to adequately debride the nonviable tissue, and utilize the simplest reconstruction available that will afford the best functional and aesthetic outcome [3, 4]. Flaps based on the superficial temporal artery form a prominent part of these algorithms [9–12]. Several reports refer to the forehead flap to reconstruct burn injuries to the midface and nose, but it is seldom applied to coverage of exposed bone of the frontal region or to provide a stable point of fixation for the larger initial STA flap. Although scalp flaps have good perfusion based on a single vessel, we also wanted to ensure that there was adequate venous drainage. The delay phenomenon, used for both flaps, is well described to augment their perfusion [13–17].

## CONCLUSION

Although a single successful free flap may result in both a shorter hospital stay and fewer procedures when reconstructing a large scalp defect, local flaps should remain a salient option when this first line procedure is contraindicated. We present a successful local scalp reconstruction in a patient with advanced age, co-morbidities and neck radiation, which precluded free tissue transfer. A local reconstruction offers advantages of replacing like-for-like tissue, lower risk of partial or complete flap loss, and reduced donor site morbidity. The delay phenomenon can be implemented to augment the local reconstruction and optimize results.
